# Emergence of *tet*(X4)-positive *Klebsiella* isolates from aquatic products in Hainan, China

**DOI:** 10.1128/aac.01618-25

**Published:** 2026-05-12

**Authors:** Yiqing Wang, Yao Wang, Weishuai Zhai, Wenyan Chen, Yang Wang, Jianzhong Shen, Chengtao Sun, Dejun Liu

**Affiliations:** 1 State Key Laboratory of Veterinary Public Health and Safety, College of Veterinary Medicine, China Agricultural University 34752, Beijing, China; 2 Technology Innovation Center for Food Safety Surveillance and Detection (Hainan), Sanya Institute of China Agricultural University 833814 , Sanya, China; University of Fribourg, Fribourg, Switzerland

**Keywords:** *tet*(X4), tigecycline, *Klebsiella pneumoniae *species complex, aquatic product, IncFII plasmid

## Abstract

The emergence of *Klebsiella pneumoniae* species complex (KpSC) with *tet*(X4) in aquatic products poses a public health risk. Among 442 aquatic products sampled in Hainan, 14 *tet*(X4)-positive isolates were identified with multidrug-resistant profiles. IncFII(pCRY)_v, a previously uncharacterized plasmid, emerged as the dominant *tet*(X4) carrier in this study, transferable by conjugation and widely present in KpSC, and particularly prevalent among clinical isolates. These findings call for One Health strategies to monitor and mitigate antimicrobial resistance in aquaculture.

## INTRODUCTION

Tigecycline is an important last-line agent (1), and its activity can be compromised by the Tet(X4) monooxygenase (2–6). Although *tet*(X4) was first described in *Escherichia coli*, subsequent studies have identified it in members of the *Klebsiella pneumoniae* species complex (KpSC), which are clinically relevant pathogens and recognized reservoirs of antimicrobial resistance genes (ARGs) (7). Since the initial detection of *tet*(X4)-positive KpSC from pork in 2021, similar isolates have been recovered from various food products in China, including meat, eggs, and vegetables (8–12). Notably, although sporadic reports have also identified the tigecycline resistance determinants *tmexCD3-toprJ1* and *tet*(X4) in aquaculture products (13), *tet*(X4)-positive KpSC have not been reported from aquatic food sources.

Between 2023 and 2024, 442 aquatic product samples comprising 211 molluscs, 149 fish, and 82 crustaceans were collected from 131 local markets across 18 administrative regions of Hainan Province, China. Fourteen *tet*(X4)-positive isolates were recovered, including 10 *K. pneumoniae* and four *K. quasipneumoniae*, originating from nine administrative regions (Fig. S1A and S1C). The detection rate was highest in fish (4.0%, 6/149), followed by molluscs (2.8%, 6/211) and crustaceans (2.4%, 2/82) (Fig. S1B).

Antimicrobial susceptibility was performed by broth microdilution, and the breakpoints for most antimicrobials were interpreted according to the Clinical and Laboratory Standards Institute (CLSI) standard (14) and the European Committee on Antimicrobial Susceptibility Testing (EUCAST) v15.0 (15) guideline. The tigecycline breakpoint (8 mg/L) was determined based on Food and Drug Administration (FDA) guidelines (https://www.fda.gov/drugs/development-resources/tigecycline-injection-products). All isolates were resistant to tigecycline and florfenicol. Of these, the minimal inhibitory concentrations (MICs) of tigecycline ranged from 8 to 32 mg/L, with an MIC_50_ of 16 mg/L (Fig. S2). In addition, most isolates were resistant to florfenicol (100%), trimethoprim-sulfamethoxazole (92.9%), ciprofloxacin (78.6%), enrofloxacin (71.4%), and ampicillin-sulbactam (50.0%). None of these isolates were resistant to meropenem, colistin, and ceftazidime/avibactam. Nevertheless, a *K. pneumoniae* strain WS_29-2 (BioSample SAMN31525422) from Thailand in the National Center for Biotechnology Information (NCBI) database carried *tet*(X4), *mcr-1.1*, and *bla*_NDM-1_, posing a threat of multiple last-line antimicrobial resistance dissemination in aquatic ecosystems (16).

**Fig 1:**
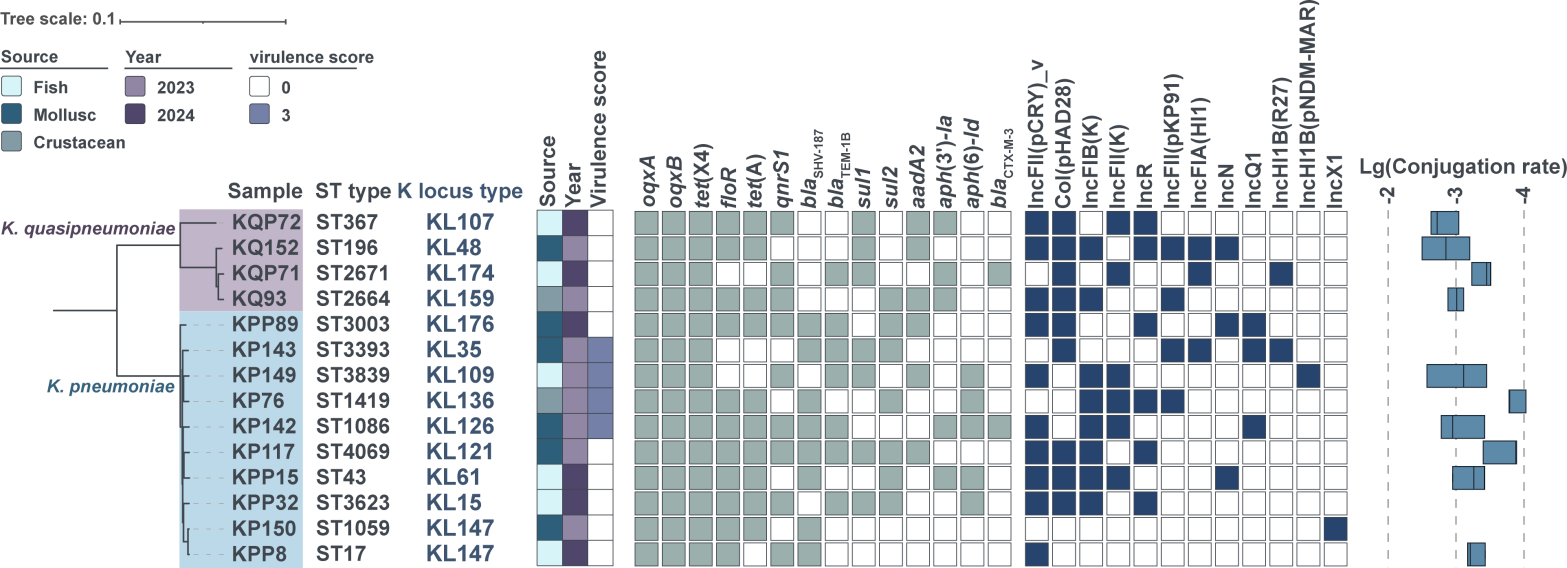
Maximum likelihood phylogenetic tree of *tet*(X4)-positive KpSC isolates collected within this study. Note: for clarity, only the most prevalent antimicrobial resistance genes and plasmid replicon types are displayed. The central line in the boxplot represents the median conjugation frequency.

To characterize their genomic features, the *tet*(X4)-positive KpSC isolates were analyzed using Abricate v1.0.1 (17) with the ResFinder (version updated 22 Mar 2024; accessed Mar 2025) (18) as well as PlasmidFinder (version updated 18 Jan 2023; accessed Mar 2025) (19, 20) database, and Kleborate v2.2.0 (21). The 14 KpSC isolates encompassed 14 different ST types and 13 K locus types (Fig. 1). Four carried a Kleborate virulence score of 3 (on a 5-point scale), but all tested negative in the string test. Despite their low virulence scores, these isolates harbored diverse antimicrobial resistance genes. In addition to *tet*(X4), more than half of these isolates carried *oqxAB* (*n* = 14), *floR* (*n* = 11), *tet*(A) (*n* = 10), *qnrS1* (*n* = 9), and *bla*_SHV-187_ (*n* = 8). These isolates shared a resistance gene profile with previously reported foodborne *tet*(X4)-positive KpSC strains characterized by frequent *oqxAB*, *tet*(A), *floR*, and *qnrS1*, suggesting shared horizontal dissemination pathways across terrestrial and aquatic food chains (8, 11).

Meanwhile, 10 of these isolates carried a plasmid replicon sharing only 81.32% nucleotide identity with IncFII(pCRY). This replicon, designated as IncFII(pCRY)_v, was the most prevalent replicon type in this study (Fig. S3). Only one *K. pneumoniae* isolate KP150 lacked IncF-type plasmids and instead carried an IncX1 plasmid. Most of the *tet*(X4)-bearing plasmids were mobilizable. Ten KpSC isolates successfully transferred *tet*(X4) to *E. coli* C600 by filter mating, with conjugation frequencies ranging from 1.4 ± 0.41 × 10^−4^ to 1.72 ± 0.73 × 10^−3^. The sizes of the conjugated plasmids determined from Illumina sequencing data and validated by reference-based genome mapping indicated seven IncFII plasmids of ~78 kb (Fig. S4A) and one IncFIA(HI1)/HI1A/HI1B plasmid of ~190 kb (Fig. S4B).

**Fig 2:**
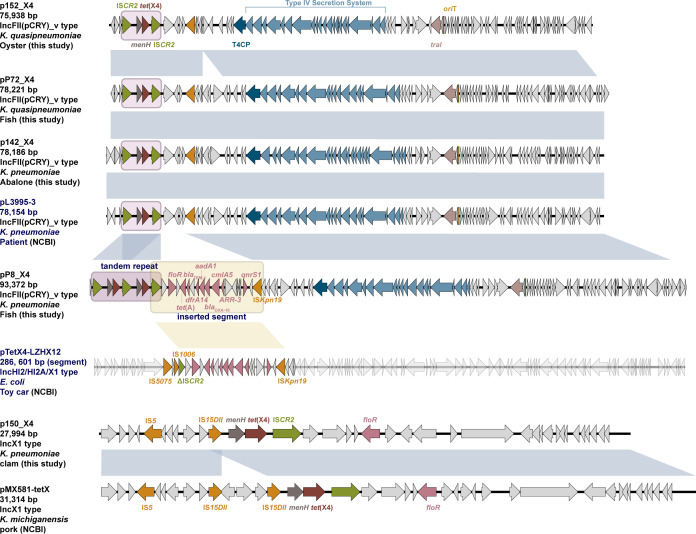
Comparison of *tet*(X4)-carrying plasmid genetic structures.

To investigate the plasmid vehicles responsible for the dissemination of *tet*(X4) within the KpSC, four isolates showing the highest conjugation rates and one isolate lacking an IncF-type replicon were selected for Nanopore MinION sequencing, assembled by trycycler v2.9.3-b1797 (22), and polished with Pilon v1.24 (23). In four isolates, *tet*(X4) was located on IncFII(pCRY)_v plasmids. Consistent with previous reports, IncFII plasmids represented one of the main *tet*(X4) conjugative vectors in *K. pneumoniae* (11, 24). Three of them exhibited highly similar structures (99.75–99.84% identity, 97–100% coverage), each containing an IS*CR2-menH-tet*(X4)-IS*CR2* segment and an entire 21,851 bp T4SS gene cluster, supporting their conjugative potential. The BLASTn analysis of plasmid p142_X4 identified three highly similar plasmids (100% coverage, 99.8% identity) recovered from pork (MW940621.1), swine (CP077430.1), and clinical (CP135167.1) KpSC isolates (Fig. 2) (20).

In contrast, the IncFII(pCRY)_v plasmid pP8_X4 (93,372 bp) from *K. pneumoniae* KPP8 shared only 87% sequence coverage with p142_X4 (99.9% identity), carrying two tandem copies of *tet*(X4) within an IS*CR2*-mediated composite structure. Additionally, a 14,202 bp segment downstream of *tet*(X4)-IS*CR2* in pP8_X4 encodes nine ARGs, including *tet*(A), *bla*_TEM-1B_, *floR*, *dfrA14*, *ant*(3)-*Ia*, *cmlA5*, *bla*_OXA-10_, *ARR-3*, and *qnrS1*. This resistant module has been frequently reported among Enterobacteriaceae. Separately, *K. pneumoniae* KP150 harbored the sole IncX1 plasmid, p150_X4, which has been widely detected in *E. coli* strains from aquatic products, human, and livestock sources (25). In this non-mobilizable plasmid, *tet*(X4) is flanked by IS*15* and IS*CR2*, distinct from IncFII(pCRY)_v but highly similar to pMX581-tetX (CP110124.1). Interestingly, comparison of *tet*(X4)-bearing plasmids from aquatic and terrestrial sources revealed no aquatic-specific backbones but strong host species associations, supporting horizontal gene transfer across ecological and host boundaries. Using the IncFII(pCRY)_v replicon as a BLASTn query (20), 95 IncFII(pCRY)_v plasmids (21,945–149,190 bp) were retrieved from the NCBI nucleotide database (as of 11 March 2025), predominantly from KpSC (90/95). These plasmids were mainly detected in humans (85/95), particularly clinical patients (55/85). With the earliest identification dating back to 2004 in Taiwan, China (CP177200.1), the IncFII(pCRY)_v plasmid has been widely disseminated in China (77/95) over the past two decades. Four of these plasmids carried *tet*(X4) and were all detected in China between 2019 and 2024, including three p142_X4-like plasmids and the fourth atypical one, pYZ2-2-tetX4 (CP127000.1).

**Fig 3:**
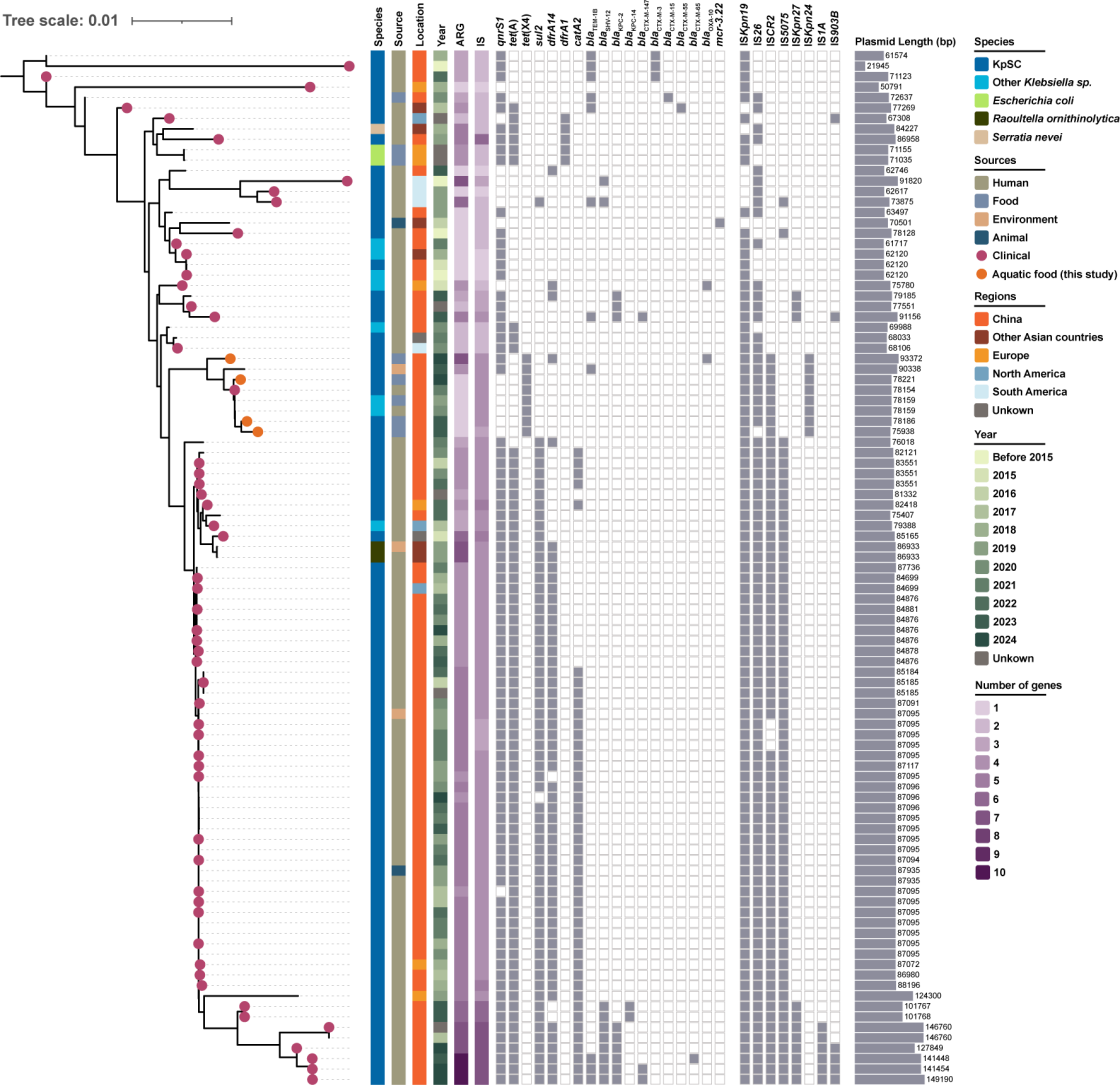
Phylogenetic tree of IncFII(pCRY)_v plasmids. Note: Only the most frequently occurring antimicrobial resistance genes and plasmid replicon types are shown. The data were collected from NCBI as of March 11, 2025.

To assess genomic relatedness, pairwise Mash distances were calculated for 99 IncFII(pCRY)_v plasmids (95 from NCBI and four from this study) using Mash v2.3 (26), revealing five distinct clusters (Mash distance < 0.01, shared hashes > 700). The largest group (*n* = 55) exhibited similar structures (Fig. S5), forming a separate subcluster adjacent to it. The phylogenetic tree built with Mashtree v1.4.6 (27) revealed differences in ARG composition (Fig. 3). In the *tet*(X4)-bearing subset, six carried only *tet*(X4), while the dominant ARGs profile in the major group (*n* = 35) featured the co-occurrence of *qnrS1*, *tet*(A), *sul2*, *dfrA14*, and *catA2*. A variety of IS elements identified by ISfinder (version updated Oct 2020; accessed Mar 2025) occurred within IncFII(pCRY)_v plasmids (28). IS*Kpn19* (*n* = 92), IS*26* (*n* = 85), and IS*CR2* (*n* = 67) were widespread. IS*Kpn19* and IS*26* commonly associated with transposition events likely facilitate the co-dissemination of ARGs, while IS*CR2* has been repeatedly implicated in *tet*(X4) mobilization (29–31). Collectively, the high diversity and abundance of IS elements may promote structural plasticity of IncFII(pCRY)_v plasmids and enhance the spread of multiple resistance determinants.

This study presents the first report of *tet*(X4)-positive KpSC isolates in aquatic food, revealing their epidemiological features, genomic traits, and transmission potential. These isolates exhibited multidrug resistance and complex genetic backgrounds, highlighting the need for enhanced surveillance and antimicrobial stewardship. The dominant *tet*(X4)-bearing plasmids in KpSC isolates demonstrate efficient transmissibility, conservative structures, and broad dissemination range, posing risks to clinical treatment. These findings emphasize the role of aquatic products as a potential reservoir and transmission route for antimicrobial resistance, reinforcing the necessity of integrated One Health strategies to curb its spread.

## Data Availability

The sequencing data of collected fourteen KpSC isolates have been submitted to NCBI under BioProject accession number PRJNA1260706.

## SUPPLEMENTAL MATERIAL

Supplemental material aac.01618-25-s0001.docx Table S1; Fig. S1 to S5.

ASM does not own the copyrights to Supplemental Material that may be linked to, or accessed through, an article. The authors have granted ASM a non-exclusive, world-wide license to publish the Supplemental Material files. Please contact the corresponding author directly for reuse.
