# Supplementary material for: Emergence of tet(X4)-positive Klebsiella isolates from aquatic products in Hainan, China
Source: Antimicrob Agents Chemother. 2026 May 12;70(6):e01618-25. doi: 10.1128/aac.01618-25 (PMC13231880; doi:10.1128/aac.01618-25)
Supplement: Supplemental material — Table S1; Fig. S1 to S5. [file aac.01618-25-s0001.docx]

**Table S1 The collection information of *tet*(X4)-positive KpSC isolates collected in this study**

| **Sample ID** | **Biosample** | **Year** | **Species** | **Location** | **Source** | **Storage temperature** | **Sample type** |
| --- | --- | --- | --- | --- | --- | --- | --- |
| KPP8 | SAMN48402419 | 2024 | *Klebsiella pneumoniae* | Ledong | fish | room temperature | farmed |
| KPP15 | SAMN48402420 | 2024 | *Klebsiella pneumoniae* | Dongfang | fish | room temperature | farmed |
| KPP32 | SAMN48402421 | 2024 | *Klebsiella pneumoniae* | Changjiang | fish | chilled storage | farmed |
| KQP71 | SAMN48402422 | 2024 | *Klebsiella quasipneumoniae* | Haikou | fish | room temperature | farmed |
| KQP72 | SAMN48402423 | 2024 | *Klebsiella quasipneumoniae* | Haikou | fish | room temperature | farmed |
| KPP89 | SAMN48402424 | 2024 | *Klebsiella pneumoniae* | Qionghai | mollusc | room temperature | farmed |
| KP76 | SAMN48402425 | 2023 | *Klebsiella pneumoniae* | Danzhou | crustacean | room temperature | farmed |
| KQ93 | SAMN48402426 | 2023 | *Klebsiella quasipneumoniae* | Danzhou | crustacean | room temperature | farmed |
| KP117 | SAMN48402427 | 2023 | *Klebsiella pneumoniae* | Qiongzhong | mollusc | room temperature | farmed |
| KP142 | SAMN48402428 | 2023 | *Klebsiella pneumoniae* | Dongfang | mollusc | room temperature | farmed |
| KP143 | SAMN48402429 | 2023 | *Klebsiella pneumoniae* | Wanning | mollusc | room temperature | wild-caught |
| KP149 | SAMN48402430 | 2023 | *Klebsiella pneumoniae* | Lingshui | fish | room temperature | farmed |
| KP150 | SAMN48402431 | 2023 | *Klebsiella pneumoniae* | Changjiang | mollusc | room temperature | farmed |
| KQ152 | SAMN48402432 | 2023 | *Klebsiella quasipneumoniae* | Ledong | mollusc | room temperature | farmed |


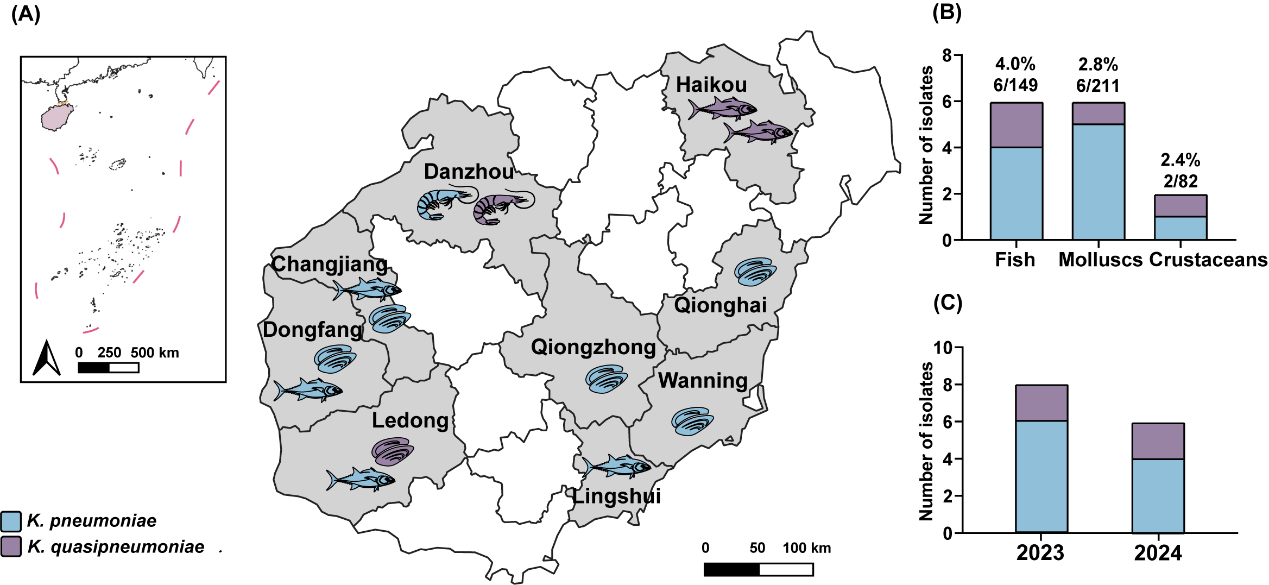


**Figure S1 The distribution of detected *tet*(X4)-positive KpSC isolates**

1. The location of collected *tet*(X4)-positive samples; (B) The aquatic products type distribution of *tet*(X4)-positive isolates; (C) The year distribution of *tet*(X4)-positive isolate


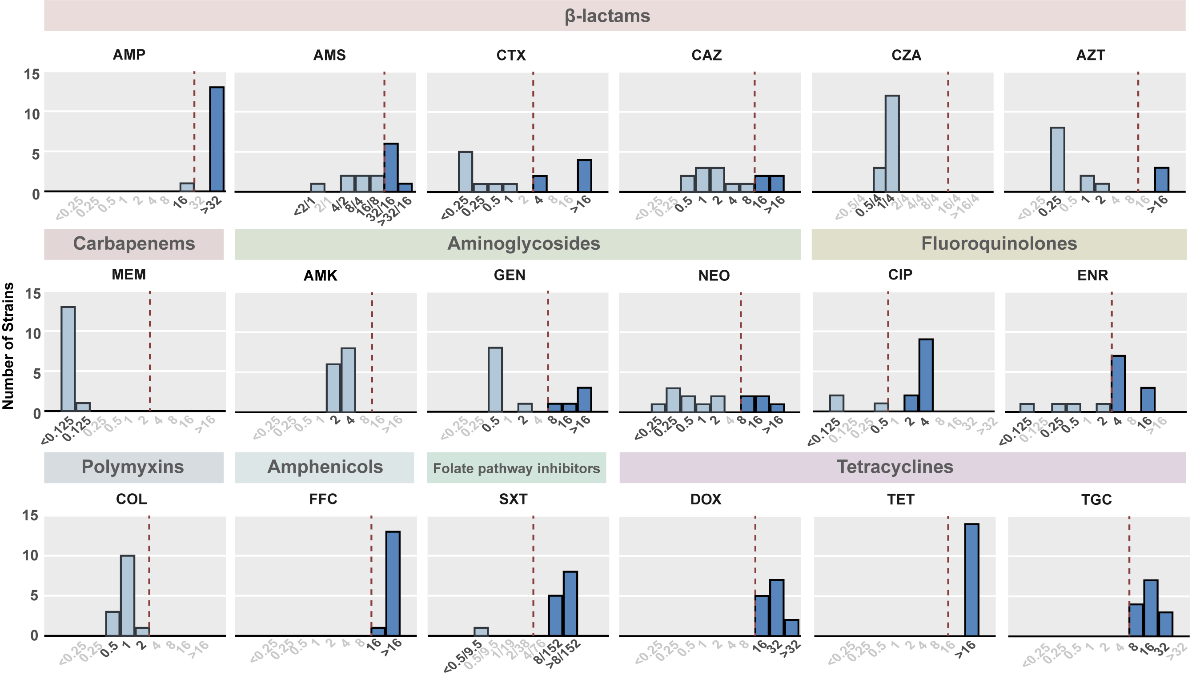


**Figure S2 The MICs of different antimicrobials shown by *tet*(X4)-KpSC isolates collected within this study (*n*=14)**

Note: 1) Ampicillin (AMP), ampicillin-sulbactam (AMS), amikacin (AMK), aztreonam (AZT), cefotaxime (CTX), ceftazidime (CAZ), ciprofloxacin (CIP), colistin (COL), ceftazidime/avibactam (CZA), doxycycline (DOX), enrofloxacin (ENR), florfenicol (FFC), gentamicin (GEN), meropenem (MEM), neomycin (NEO), trimethoprim-sulfamethoxazole (SXT), tetracycline (TET), and tigecycline (TGC); 2) The red dash lines represent the level of resistant breakpoint; colons in dark blue represent resistant strains.


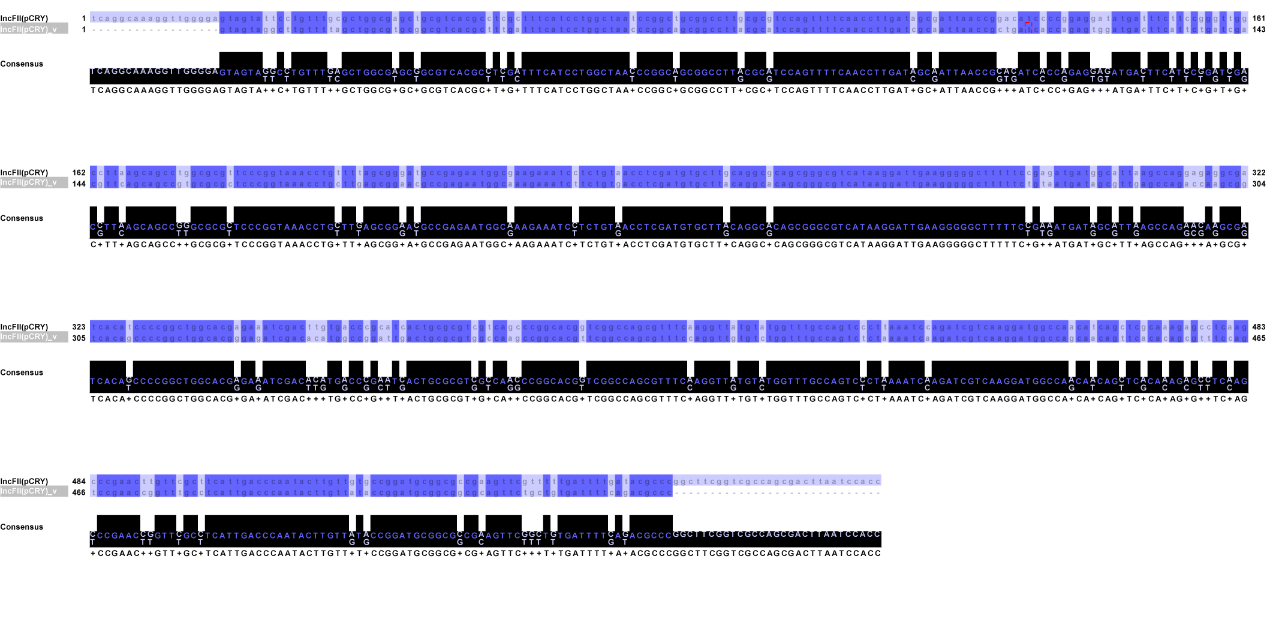


**Figure S3 Alignment of IncFII(pCRY) plasmid replicon and its variant**

Note: Dark colors denote conserved regions, while lighter colors highlight sequence variations.


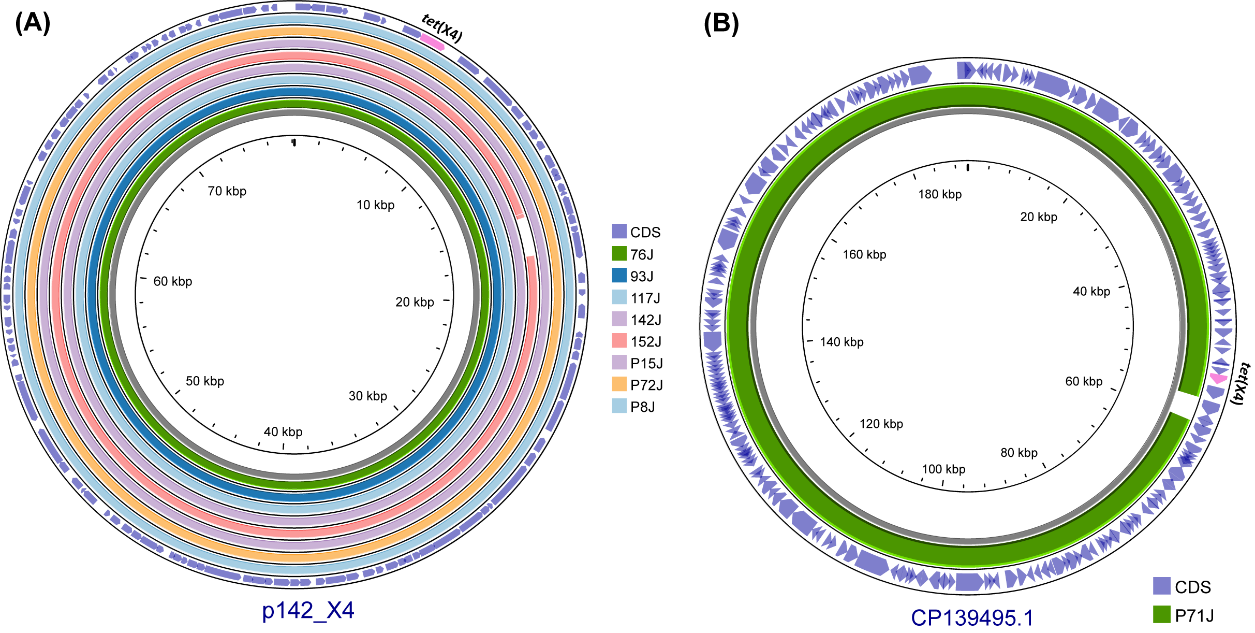


**Figure S4 The plasmid structures of conjugated plasmids**

(A) IncFII(pCRY)_v-type conjugated plasmids; (B) IncFIA(HI1)/HI1A/HI1B-type conjugated plasmid

Note: One transconjugant was not successfully recovered during revival, and thus only nine transconjugants were subjected to mapping analysis.


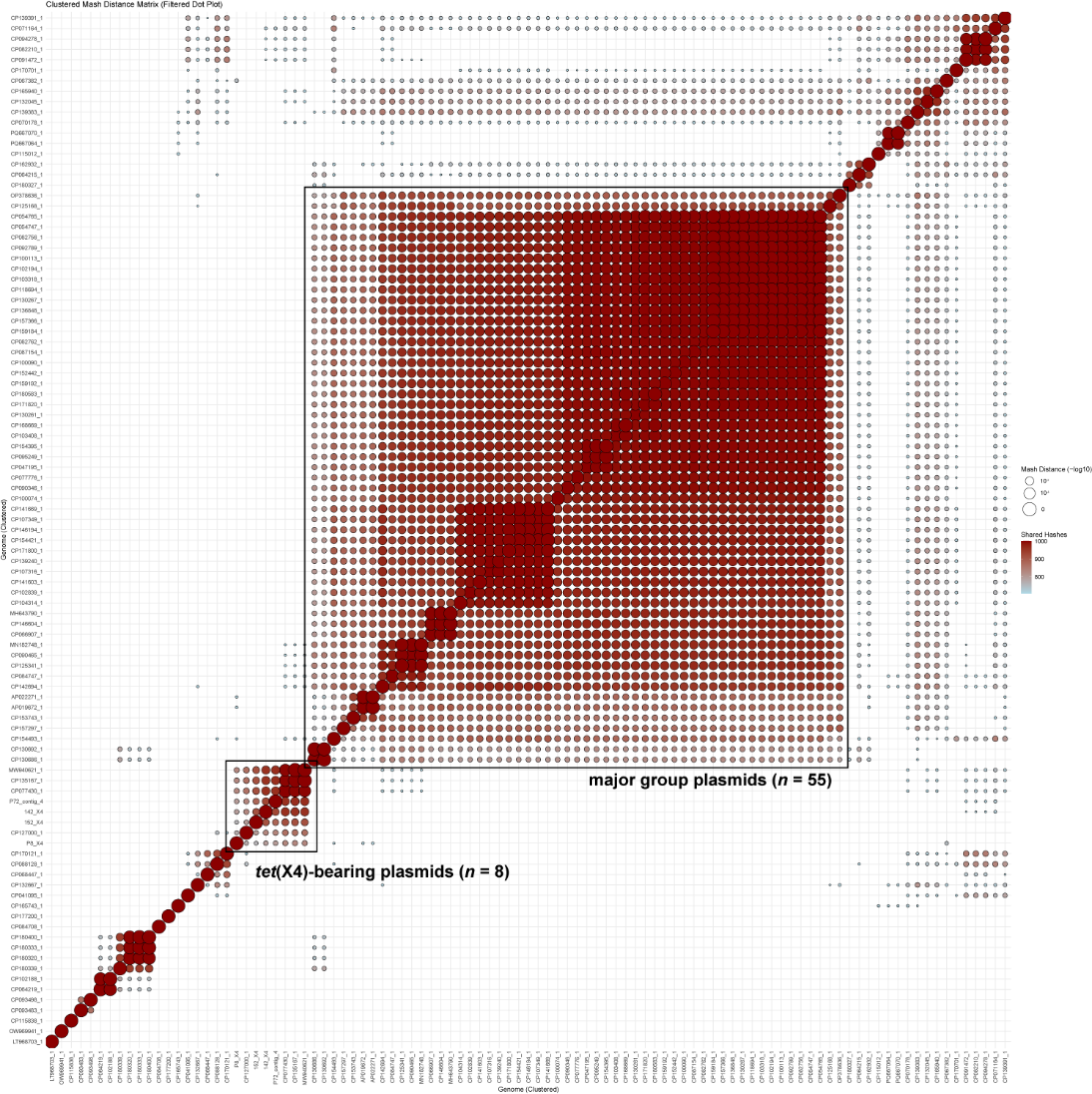


**Figure S5 The mash distances of IncFII(pCRY)_v plasmids**
